# Impact of soil salinity on structural attributes and above ground biomass carbon in a mangrove community of a Colombian Caribbean Coast

**DOI:** 10.1038/s41598-025-10278-6

**Published:** 2025-08-06

**Authors:** Rodrigo Rodríguez-Reales, Juan Pablo Gómez, Jimena Bohórquez-Herrera, María Cristina Martínez-Habibe

**Affiliations:** 1 Chemistry and Biology Department, Universidad del Norte, Barranquilla, Colombia; 2 Faculty of Exact and Natural Sciences, Biology Program , Universidad de Cartagena, Cartagena, Colombia

**Keywords:** Mangrove ecosystems, Above ground biomass carbon, Carbon sequestration, Tree community composition, Soil salinity, Environmental factors, Ecology, Environmental sciences

## Abstract

Mangrove forests are known for their exceptional carbon storage capacity, but the influence of environmental factors on this service remains understudied. This study examines how environmental conditions shape tree community composition and carbon storage in Mallorquin Swamp, an urban mangrove ecosystem in Barranquilla, Colombia. We assessed tree composition, vegetation structure, soil pH, and salinity across 18 circular plots in areas of Low, Medium, and High salinity. Above ground biomass (AGB) and carbon stock were estimated using allometric equations and wood density databases. Our findings revealed significant salinity differences among sampling areas, especially during the dry season, while soil pH showed minimal variation. *Avicennia germinans* was dominant in Low salinity areas, *Laguncularia racemosa* in Medium salinity areas, and *Rhizophora mangle* in High salinity areas. Trees in Low salinity zones were notably taller and larger, contributing to significantly higher carbon stock (4098.6 Mg C) compared to Medium (104.6 Mg C) and High (1761 Mg C) salinity areas. These results underscore the importance of local environmental factors, particularly salinity, in shaping mangrove structure and carbon dynamics. Identifying such patterns is vital for guiding conservation efforts and carbon policies, particularly in urban and climate-sensitive areas, where focused management can strengthen mangrove resilience and carbon storage.

## Introduction

Mangroves are widely recognized as the main carbon sinks among tropical forests^1,2^, contributing to climate change mitigation^3–5^. Despite representing only 0.81% of the world’s tropical forests^6,7^, mangroves store up to five times more carbon per hectare than any terrestrial forest^1^. However, knowledge about carbon capture and storage in this type of ecosystem remains limited in several countries across Africa, South America, and Southeast Asia^8^.

The capacity of mangroves to function as effective carbon sinks is intricately linked to various abiotic factors. Abiotic factors, such as hydroperiod and topography, have an impact on mangrove species composition and richness^9,10^. These characteristics influence physical and chemical properties like salinity and soil nutrient availability, which are mediated by pH^10,11^. Consequently, these factors impact the ability of mangrove species to photosynthesize and absorb carbon dioxide from the atmosphere^12–15^. It is well established that large-diameter trees play a significant role in contributing to above ground biomass (AGB) stocks. However, salinity has a considerable negative impact on AGB stocks. As salinity levels exceed 25 PSU, the presence of large-diameter trees diminishes, reducing their contribution to AGB stocks^16^. Furthermore, areas with moderate or low salinity may support a greater diversity of species adapted to those conditions^17^, while high salinity zones exhibit lower diversity and complexity^18^. These findings demonstrate that both salinity levels and the predominance of specific tree species impact the distribution of AGB stocks and the species diversity in mangrove ecosystems.

Salinity is a key environmental factor influencing the distribution, productivity, and structure of mangrove forests, as it affects plant water uptake and photosynthetic capacity^19^. In urban settings, hydrological alterations, often driven by infrastructure development, can significantly modify salinity regimes, thereby impacting mangrove community composition. For instance, studies in Puerto Rico have shown that mangrove species composition shifts along urban gradients, with changes in porewater salinity strongly associated with the degree of urbanization^20^. Similarly, a study in Hong Kong’s urban mangroves found that increased salinity levels were linked to reduced canopy height, species diversity, and carbon storage^21^. Comparable impacts of increased salinity on mangrove structure have also been documented in non-urbanized deltaic systems, such as the Sundarbans in Bangladesh^22^. These deltaic dynamics resemble those found near the mouth of the Magdalena River in the Caribbean Coast of Colombia, where the Mallorquin Swamp forms part of a complex estuarine system subject to fluctuating salinity and human interventions^23^.

In Colombia, few quantitative estimates of carbon storage in mangroves exist for the Caribbean Coast, and none for the department of Atlántico^24–28^. Abiotic factors that influence mangrove forests are highly variable along the coastline. For example, closer to the mouth of large rivers such as the Magdalena, water salinity is low and coastal erosion is high. This lack of information makes it difficult to obtain large-scale carbon data, which would support national initiatives for mitigating climate change^27^.

The Mallorquin Swamp is one of the places in Colombia with the largest extension of urban mangrove forests, located off the coast of Barranquilla, the largest city in the Caribbean region of Colombia. Abiotic conditions are highly variable due to the construction of the “Tajamar” training wall between 1922 and 1935^29^. This construction significantly altered the hydrodynamics of the estuary and Magdalena River delta, leading to noticeable changes in the morphology of the coastline, and a sustained loss of approximately 650 hectares of wetlands to the west of Tajamar^29,30^. Currently, its mangrove forests are composed mainly of four species: *Avicennia germinans (L.), Rhizophora mangle L., Laguncularia racemosa (L.) C.F. Gaertn., and Conocarpus erectus L*^31,32^*.*

Over 20,300 inhabitants live around the Mallorquin Swamp. Illegal housing developments with inadequate access to healthcare, education, and employment opportunities fuel the population growth in this area^33^. The mangrove ecosystem in the area is severely degraded, primarily because of human activities such wastewater discharge, deforestation, illegal land occupations, and solid waste disposal^34^. There have been vast changes in water flux into the swamp that potentially have altered its pH and salinity, among other abiotic factors. For example, the isolation of the swamp from the Magdalena River to improve its navigability , has significantly reduced the fluxes of sediment and freshwater to the mangrove ecosystem. As a result, after the Tajamar was built, these fluxes that had formerly enriched the delta and the“Mallorquin Swamp”lagoon system to the west, were predominantly shifted offshore^30,35^.

Previous studies in the Mallorquin Swamp have primarily focused on mangrove forest composition and ecosystem quality. INVEMAR^31^ (the Colombian Institute of Marine and Coastal Research) has described the area as having high salinity and prolonged droughts, which have nearly eliminated *R. mangle*, allowing more salt-tolerant species like *A. germinans* and *L. racemosa* to dominate. Environmental challenges such as low oxygen levels, excessive organic matter, and fecal coliform contamination have been documented^36^, impacting aquatic life and overall ecosystem health. Heavy metal contamination also poses a significant threat. Universidad del Norte^37^ reported cadmium, chromium, and lead in the swamp, potentially linked to leachates from the nearby ‘Las Flores’ landfill which operated from approximately 1970 to 1990^38^. Fuentes-Gandara et al.^39^ found high concentrations of zinc, copper, cadmium, and mercury near the landfill, which may exceed mangrove remediation capacity and reduce forest biomass^40,41^. These pollutants, combined with physicochemical stressors such as salinity and pH fluctuations, exacerbate the pressures on this ecosystem.

Anthropogenic activities are well-known to significantly influence soil quality, which in turn affects the structure, productivity, and spatial distribution of mangrove species^11^. Despite the ecological importance of mangroves and the challenges facing the Mallorquin Swamp, to date, no studies have examined how key soil physicochemical parameters, such as salinity and pH, influence the structural attributes and AGB of this mangrove community. To bridge this knowledge gap, our study focuses on analyzing the relationship between soil abiotic factors and the diversity, structural composition, and AGB of mangrove forests in the Mallorquin Swamp, located in Barranquilla, Colombia.

Given Mallorquin’s disturbance history, we expect to observe differences in the composition and structure of mangrove forests that result in differences in carbon content of AGB across the swampland. Specifically, we expect that the area closer to freshwater influx such as the Magdalena River will be composed of taller and bigger trees that store the largest amount of carbon. In areas in which there is higher influence of salt water or freshwater influx has been decreased, we expect a less complex mangrove forest with a lower carbon stock. Understanding how these changes in abiotic variables influence the mangrove forest are key to predicting the conservation and restoration trajectories and accurately estimate the carbon stock of this important urban mangrove.

## Methods

### Study area

**Fig. 1:**
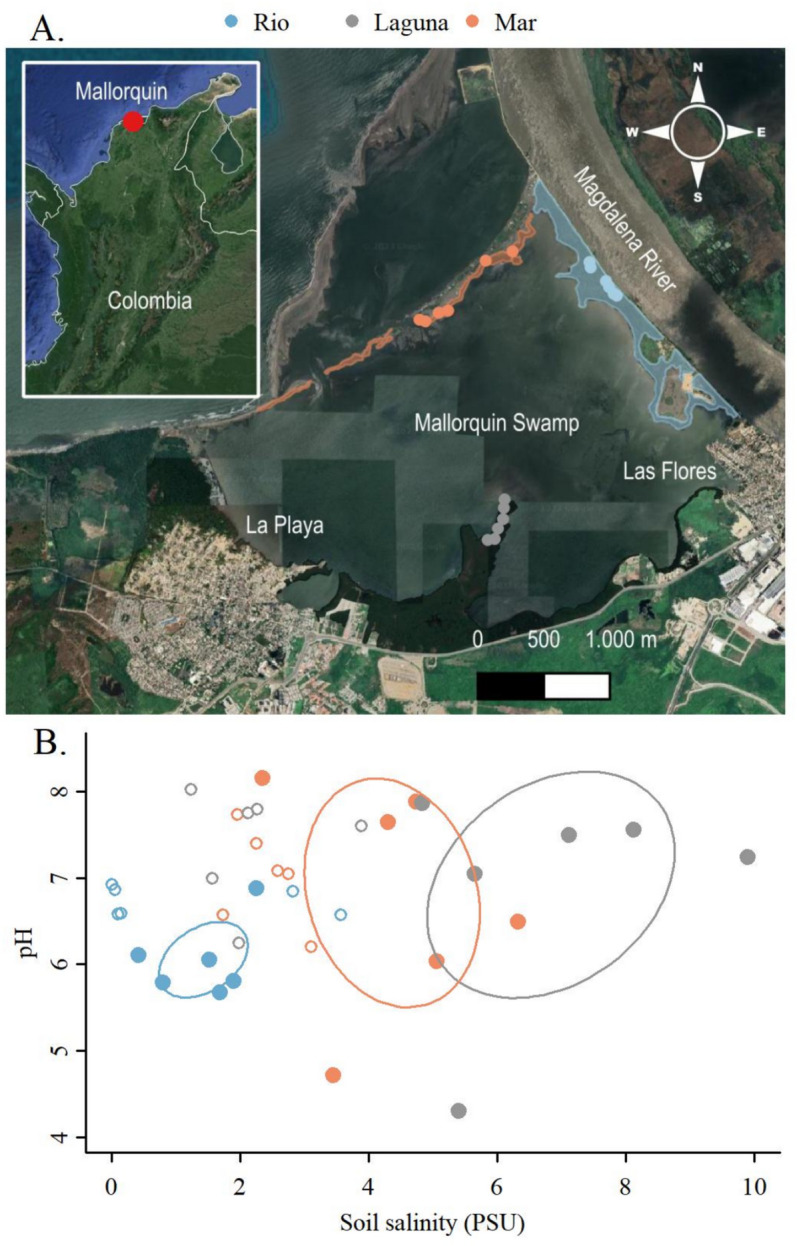
Map of the urban swamp of Mallorquin showing the location of the sampling stations (colored polygons) and circular plots (colored solid circles) where physicochemical and vegetation variables were collected (**A**) and the measurements of soil water and salinity (**B**) during the rainy (open circles) and dry seasons (solid circles). The colored polygons are ellipses describing the grouping based on the centroid and one standard deviation of the physicochemical measurements on each plot during the dry season. Contrary to our expectations, the highest salinity region was Laguna, followed by Mar and finally the least salty region was Rio. Polygons show each station´s total area. PSU = Practical Salinity Units. The map was generated by the authors using QGIS version 3.26.2 ‘Buenos Aires’ (https://www.qgis.org). The base image was imported via the QuickMapServices plugin version 0.19.29, using Google Satellite imagery. The map was then exported as an image and combined with the plot using R version 4.2.2 for Windows (https://www.r-project.org).

This research was carried out in the mangrove forests of the Mallorquin Swamp (650 ha), located in the north of the city of Barranquilla, in the Atlántico department, at the Colombian Caribbean Coast (11°2′47.04″ N, 74°50′48.12″ W) (Fig. 1). The Mallorquin Swamp is a coastal lagoon of great relevance for the region since it was declared a RAMSAR Site ^42^ and comprises one of the largest extensions of mangrove forests in the Atlántico department^43^. Rainfall is extremely seasonal, with two drought periods, the first one between December and March, and the second one between July and August. The months from September to November experience the highest levels of precipitation. The mean annual temperature is 28 °C, with a mean annual relative humidity of 80% and an annual precipitation total of 900 mm^44^.

We established three sampling stations located within the Mallorquin Swamp with varying fresh and saltwater influence (Fig. 1). The Rio station (33.6 ha), located in the northeast area of Mallorquin, was characterized for having mostly fresh water through a direct connection with the Magdalena River through three concrete box culverts. The Laguna station (74.8 ha) was set in the area known as Punta de Felix and has the influence of both the Magdalena River and the Caribbean Sea, making its waters an almost even mixture of fresh and salt water. Finally, the Mar station (9 ha), located in the north end of Mallorquin was characterized by having greater influence of the Caribbean Sea due to its proximity to the Puerto Mocho Beach, making its waters mostly salty.

### Field measurements

Within each sampling station, we located six circular plots with a radius of 7 m (153 m^2^) which are ideal for estimating the structure, biomass, and carbon stock of mangrove forests^2^. Plots were located along a ~ 350 m transect to encompass the high natural variability in mangrove forests^2^. This methodology has been previously used for the estimation of carbon stocks in mangroves across the neotropics and is particularly designed to capture the high heterogeneity of this type of habitat^45,46^. We measured the diameter at breast height (DBH), total height (TH), and basal area (BA)—the cross-sectional area of a tree trunk at breast height—for all trees of the three species with a DBH ≥ 5 cm in each plot. We also computed the standard deviation (SD) of the height of all trees as a metric of vertical vegetation structure. The species dominating the ecosystem were *Avicennia germinans*, *Rhizophora mangle* and *Laguncularia racemosa*. The formal identification of the plant material was conducted in the field by María Cristina Martínez-Habibe, a botanist and taxonomist, based on morphological characteristics. Voucher specimens were not collected due to the non-destructive nature of the sampling protocol; however, the geographic coordinates of each individual tree were recorded for verification and traceability. To measure soil’s salinity and pH, we took a soil sample in the center of the plot once during the rainy season of 2020 and one during the dry season of 2021. The samples were taken to the environmental engineering laboratory at Universidad del Norte and analyzed using a Multi 3420 m by WTW. We took two soil samples for each plot and each season. In addition, pH and salinity levels were measured three times for each soil sample taken. Salinity was expressed as Practical Salinity Units (PSU).

### Carbon estimation of Above Ground Biomass (AGB)

AGB for each tree in a plot was estimated using the mangrove forest specific allometric equation in Chave et al.^47^: Biomass (Kg) = 0.0509 × ρ x DBH^2^ x TH, where 0.0509 is a multiplicative coefficient, ρ is the species-specific wood density (g/cm^3^), DBH is the diameter at breast height (cm) and TH is the total height (m). The specific wood density of *Avicennia germinans* (0.7764 g/cm^3^), *Rhizophora mangle* (0.8977143 g/cm^3^) and *Laguncularia racemosa* (0.61 g/cm^3^) were obtained from the Global Wood Density Database^48,49^. For *Rhizophora mangle*, only the main trunk DBH at 1.3 m was measured, excluding prop roots initiating above this height, following standard practice and ensuring consistency with the allometric model. Carbon stock was estimated as 0.5*Biomass^2^. Carbon contents in Kilograms were converted to Megagrams (Mg).

### Statistical analysis

To evaluate the effect of salinity and pH on compositional and structural characteristics of mangrove forests, we took a multilevel approach in which we first evaluated differences in abiotic characteristics among plots, then evaluated changes in mangrove forest composition and structure, and lastly, evaluated differences in aboveground carbon stock. First, we evaluated the differences in physicochemical variables among plots using an analysis of similarity (i.e., ANOSIM) based on the bivariate Euclidean distance matrix constructed using soil pH and salinity^50^. We also tested for univariate differences among stations to separate the effects of pH and salinity independently. We grouped plots based on the station they were established on, Rio, Mar or Laguna. The significance of the ANOSIM was estimated using 1000 randomizations of the physicochemical distance matrix. We ran three separate ANOSIM, the first one using the average of the measurements taken during the dry and rainy season and two different ones including measurements during the dry and rainy seasons separately.

Second, we used a non-metric multidimensional scaling analysis based on the Bray–Curtis dissimilarity index to evaluate the differences in plant community composition among plots and stations and generalized linear models assuming gamma distributed error to compare tree height, size and forest vertical stratification, carbon stock, and diversity using Hill numbers (q0, q1 and q2). Difference in q0 among plots and stations was modeled assuming a Poisson distributed error. BA was used as abundance metric for each species for computing diversity indices. Hill numbers correspond to species richness (q0), the exponent of the Shannon diversity index (q1) and the inverse of the Gini-Simpson index (q2)^51,52^. These three dimensions along with the Bray–Curtis dissimilarity index properly describe community dynamics by measuring alpha and beta diversity and patterns of evenness and dominance^51,53^. We estimated the uncertainty in the coefficients and the predictions using parametric Bootstrap by simulating 1000 datasets of the dependent variables using the best fitting model and re-estimating the parameters and predicted values. The confidence intervals for the models and predictions are given by the 2.5 and 97.5 quantiles of the distribution of the estimations with the 1000 simulated datasets. For each of the response variables, we compared a null model with intercept only with a model including station as independent variable using the Bayesian Information Criterion (BIC). We assumed strong evidence in favor of the model with the lowest BIC when the difference in BIC between the null and alternative model was larger than 4 BIC units^54^.

## Results

### Differences in salinity and pH

The analyses of similarity based on the bivariate Euclidean distance matrix showed significant differences in salinity and pH among stations during both the dry (ANOSIM statistic R = 0.6, p = 0.001) and the rainy (ANOSIM statistic R = 0.3, p = 0.02) seasons and across the year (ANOSIM statistic R = 0.41, p = 0.002); with much stronger differences during the dry than during the rainy season (Fig. 1).

### Salinity and pH variability among stations

The univariate analysis showed that the stations vary consistently in salinity and much less according to pH as revealed by the R statistic of the ANOSIM (Salinity ANOSIM statistic R = 0.4, p = 0.001; pH ANOSIM statistic R = 0.25, p = 0.02). The station with the highest levels of soil salinity was Laguna, followed by Mar and finally the Rio station (Fig. 1). From now on we will refer to the Rio, Mar and Laguna stations as Low, Medium and High salinity stations respectively. Although the ANOSIM showed significant differences in soil pH consistent with station groups, visually these differences are much less pronounced than for salinity (Fig. 1).

### Community composition across salinity levels

**Fig. 2:**
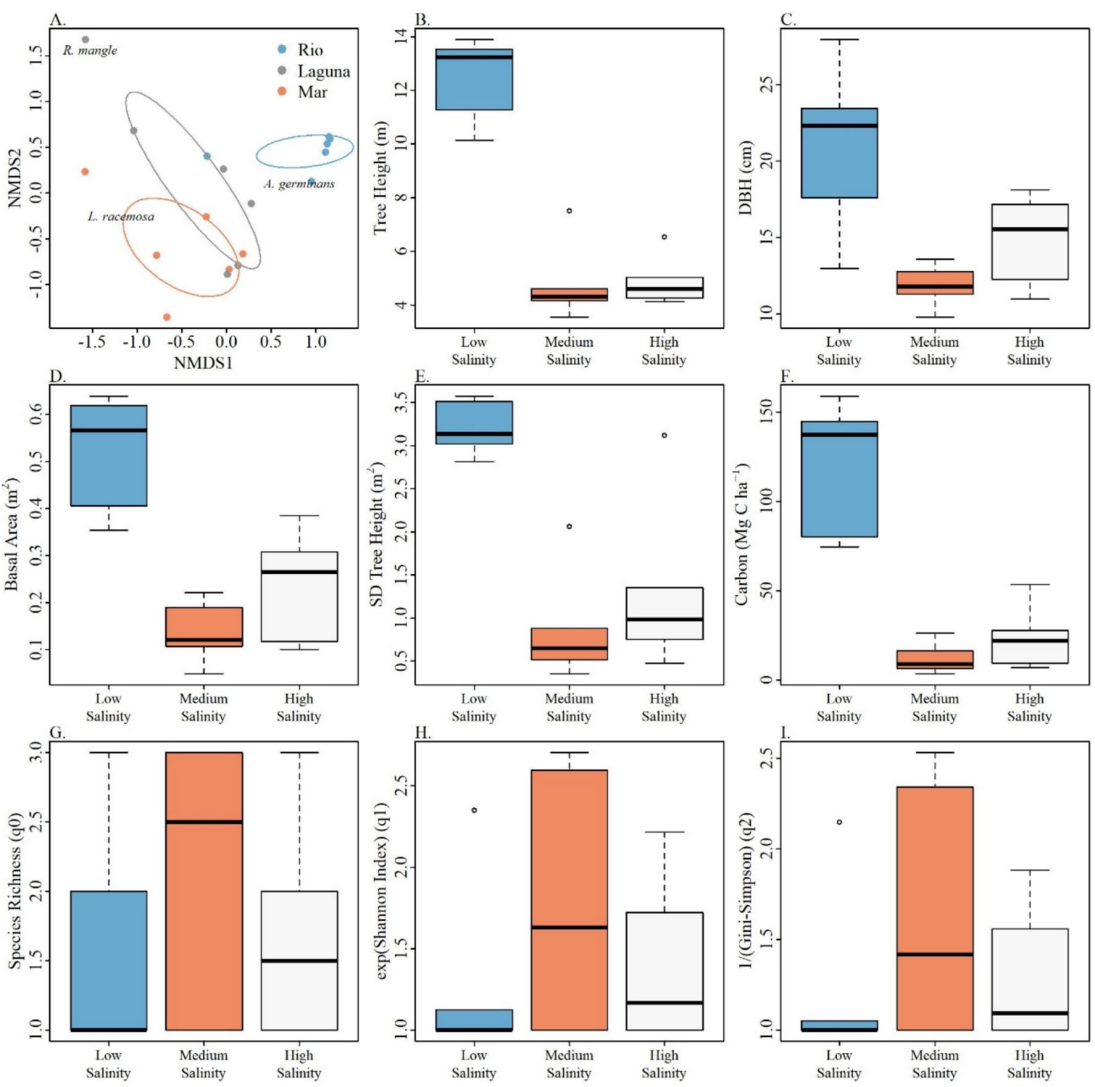
Community composition and vegetation structure of the plots in each of the three stations. Panel **A**. shows the results of a non-metric multidimensional scaling analysis with the position of each of the plots based on their compositional similarity. The closer the two points are together, the more similar they are. Species in the figure show to which plots these species are mostly associated. Panels **B**—**G** are boxplots showing the differences in tree height, Diameter at Breast Height (DBH), tree abundance (Basal Area), vertical vegetation structure (SD tree height), carbon stock (Mg) and diversity by station, respectively. Low Salinity corresponds to Rio, Medium Salinity to Mar and High Salinity to Laguna. Colors in the boxplots follow the colors in Panel A. In the boxplots, the upper and lower limits of the box represent the 25^th^ and 75^th^ percentiles and the solid line within the plot shows the median of the distribution of the variable. The upper and lower limits of the error bars show the 10^th^ and 90^th^ quartile. Finally, points outside the box and the error bars show outlier observations from the distribution.

Community composition was different among stations. While plots in the Low salinity station were mostly associated with *Avicennia germinans*, *Laguncularia racemosa* was mostly associated with Medium salinity plots (Fig. 2A). This is evident by the position of the species scores in relation to the plot scores. The closer a species score is to a plot score, the higher the association of that species with that plot. The plots in High salinity were the most heterogeneous showing differences in composition, some of them similar to plots in Medium salinity and others to the Low salinity stations and one plot mostly composed of *Rhizophora mangle* trees (Fig. 2A).

### Vegetation structure and carbon stock

We found significant differences in tree height, abundance (measured as BA), vertical vegetation structure and carbon stock among stations but not in tree diversity (Fig. 2B-E, Table 1). In general, the Low salinity station was composed of significantly taller and bigger trees and a significantly higher vertical stratification resulting in a significantly larger carbon stock, compared to the plots in the Medium and High salinity stations. The latter two stations showed significant differences only in tree BA in which the Medium salinity station had larger trees than the High salinity station. Given the area of the mangrove forests in each station, we estimate that Carbon stock is 4098.6 (2092–6105.3) Mg C in the Low salinity station, 104.6 (53.4–155.7) Mg C in the Medium salinity station and 1761 (898.9–2623) Mg C in the High salinity station.

**Table 1:** Model estimates and their confidence intervals in parenthesis showing significant differences in tree height, Tree size, forest vertical structure, carbon stock and diversity. ΔBIC shows the difference in Bayesian information criterion between the model with station as independent variable and the null model with no explanatory variable. ΔBIC < 4 units indicate strong evidence in favor of the alternative model suggesting differences in dependent variables among stations. Letters in the super index show groups of stations with significant and non-significant differences in vegetation structure. The groupings were based on the overlap of the 95% Confidence Intervals of the mean.

	**Rio**	**Mar**	**Laguna**	**ΔBIC**
Tree height (m)	12.6 (10.4–14.7)^a^	4.9 (4–5.7)^b^	4.7 (3.9–5.5)^b^	−32
DBH (cm)	21.1 (17.9–24.4)^a^	11.9 (10–13.7)^b^	14.9 (12.6–17.2)^b^	−12.3
Basal area (m2)	0.5 (0.4–0.7)^a^	0.2 (0.2–0.3)^b^	0.1 (0.1–0.2)^c^	−14.2
SD Tree height (m2)	3.2 (1.6–4.8)^a^	1.3 (0.7–1.9)^b^	0.9 (0.4–1.3)^b^	−10.2
Carbon stock (Mg)	1831.6 (934.9–2728.3)^a^	174.8 (89.2–260.4)^b^	353.1 (180.2–525.9)^b^	−22.1
q0	1.5 (0.5–2.5)^a^	2.2 (1–3.3)^a^	1.7 (0.6–2.7)^a^	5
q1	1.2 (0.8–1.7)^a^	1.4 (0.9–1.8)^a^	1.8 (1.2–2.3)^a^	3
q2	1.2 (0.8–1.6)^a^	1.6 (1.1–2.1)^a^	1.3 (0.9–1.7)^a^	3

## Discussion

In this study, we attempted to evaluate the effects of salinity and pH on mangrove forest community composition and carbon storage in an urban swampland in Northern Colombia. Our results show that salinity conditions and pH vary significantly within the Mallorquin Swamp, influencing tree structure and carbon storage. Nonetheless, salinity differences are much more pronounced than soil pH differences. The differences in soil salinity were more pronounced during the dry season, potentially imposing strong limitations for growth and recruitment of mangrove trees. Furthermore, we found strong differences in community composition, vegetation structure and carbon storage among areas with different levels of salinity. Specifically, we found that salinity negatively influenced mangrove tree size and carbon stock but not diversity. In fact, although the relationship was not significant, tree diversity was lower in Low salinity soils compared to medium salinity soils.

The observed significant differences in salinity and pH among stations during both the dry and rainy seasons, as well as across the year, highlight the dynamic nature of the Mallorquin Swamp in Barranquilla, Colombia; as it has been reported in other mangrove ecosystems^55,56^. These variations are attributed to factors such as temperature, salinity, and rainfall, which can impact the water quality and sediment characteristics of the mangroves^55,56^. The dry season is related to lower water levels and higher salinity, whereas the rainy season delivers higher water levels and decreased salinity^55,56^. The variations in physicochemical properties between the dry and rainy seasons indicate that environmental conditions affect mangrove trees periodically, influencing leaf chlorophyll content and morphology^57^. Notably, during the rainy season, chlorophyll concentration increases in mangroves under poor conditions, whereas healthy plants exhibit little fluctuation across seasons^57^.

The observed variation in community composition among the Low, Medium, and High salinity stations highlights the influence of soil salinity on the distribution of mangrove species within the Mallorquin Swamp*. Avicennia germinans*, the black mangrove, is well-adapted to low salinity environments, as evidenced by its dominance in the Low salinity station. This characteristic may be due to the seasonal supply of fresh water and, consequently, nutrients^58,59^ as has been demonstrated in other mangroves on the Colombian Caribbean Coast^60,61^. Furthermore, *A. germinans* has anatomical and morphological adaptations that allow it to tolerate and grow in a wide range of salinity^62^ that could reach up to 90 PSU^63^. In response to high salinity, the wood anatomy of *A. germinans* exhibits increased vessel wall thickness, vessel frequency, and fiber lumen diameter, balancing hydraulic efficiency and mechanical stability^64^. Morphological adaptations include pneumatophores, which are specialized roots that aid in gas exchange, and thick leaves with salt glands for salt excretion^65^. However, López et al^66^ showed that *A. germinans* trees decrease significantly in TH and BA at salinity levels between 10 and 30 PSU. Also, the production of new leaves and the leaf specific area of *A. germinans* are reduced as salinity increases^67^.

In contrast, the Medium salinity station was dominated by *Laguncularia racemosa*, showcasing its remarkable adaptability to moderate saline conditions. Consistent with Torres-Duque^68^and INVEMAR^69^, a decrease in the abundance of *A. germinans* along with an increase in *L. racemosa* indicates areas undergoing regeneration. This pattern reflects with the rapid colonization capacity of *L. racemosa* during secondary succession processes in mangrove ecosystems, as described by Soares et al.^70^ and De Sousa et al.^71^. The rapid colonization of *L. racemosa* in new territories is linked to its stomatal traits, with a greater number of smaller stomata enhancing gas exchange and photosynthetic rates. This adaptation allows for increased nutrient uptake and faster growth compared to other mangrove species^72^.

Interestingly, we observed no significant effect of salinity on tree diversity, though diversity appeared slightly lower in the Low salinity station compared to the Medium salinity station. This contrasts with findings by Chandrasekara et al.^73^ who suggested that salinity gradients often correlate with shifts in species composition and diversity due to differential salt tolerance among mangrove species. Our results may reflect site-specific conditions or the dominance of salt-tolerant species like *Avicennia germinans* and *Laguncularia racemosa*, which thrive across a range of salinity levels^62,74^. Additionally, other environmental factors, such as nutrient availability, ground elevation, or hydrological dynamics, could also influence the observed patterns but were not assessed in this study.

Although the High salinity station exhibited a heterogeneous composition, *Rhizophora mangle* was the most dominant and abundant in this station (Fig. 2A), suggesting a certain degree of tolerance to higher salinity, challenging traditional associations of this species with lower salinity environments^75^. These results suggest that *R. mangle* thrives along tidally influenced coasts due to effective propagule transport by tidal currents^76,77^. This species establishes itself on the forest edges, occupying perpetually flooded areas^78^. It possesses an extensive internal airflow system, where air travels through stem aerenchyma to stilt roots, delivering oxygen to the growing root sections in anoxic substrates^79^.

The differences in salinity across the stations in Mallorquin are mainly created by anthropogenic causes due to the construction of the training wall for the Barranquilla port which changed the incoming fresh water dynamics from the Magdalena River. Such changes date to the early 1900 s such that there has been opportunity for rapid adaptation of these mangrove species to high salinity conditions. This could explain why the high salinity areas still retain some diversity despite the harsh environmental conditions. Such adaptation has been shown to happen in other tropical mangroves^80^. Another alternative explanation to why the high salinity station retains some diversity is that salinity is not extreme enough to cause massive mangrove death^81^. In our high salinity station, the salinity was most extreme during the dry season reaching values up to 10 PSU. Nonetheless, it has been reported that mangrove trees start experiencing problems with salt regulation when salinity reaches 40 PSU^81^.

The presence of significantly taller and bigger trees, as well as a larger carbon stock in the Low salinity station, is supported by several studies. Specifically, Rahman et al.^82^ found that salinity can influence carbon stock in the Sundarbans mangrove forest, the world’s largest mangrove forest, with the freshwater zone showing the highest stock. Moreover, Ahmed et al.^16^ documented that soil salinity had a strong negative effect on growth dominance coefficient, AGB, and AGB gain. An alternative explanation for this pattern is that the forests in the low salinity area tend to be the oldest.

One pivotal aspect contributing to the distinctive characteristics of the Low salinity station is its direct association with the Magdalena River, as documented by Garcés-Ordóñez et al.^32^. This influence emanates from the regulated flow of freshwater facilitated by three box culverts, specialized hydraulic structures designed for the controlled conveyance of liquids. The consistent freshwater input from the Magdalena River shapes the unique environmental conditions of the Low salinity station, fostering conditions conducive to the observed robust vegetation and enhanced carbon stock.

In contrast, the High and Medium salinity stations are characterized by having more saline soils and a higher pH, which could be due to their proximity to the saltwater of the Caribbean Sea and brackish waters of the swamp. These waters tend to have a high pH due to the acid–base balance between atmospheric carbon dioxide absorption and the involvement of calcium carbonate from the leaching of limestone rocks^83,84^, which are common in the area’s geomorphology^37,85,86^. These characteristics could have impacted negatively the heights, BA, and carbon storage of the mangroves at the High and Medium salinity stations (Fig. 2). This may be explained by an increase in salt concentration which reduces the soil water potential and makes it difficult for plants to absorb water^12,87^; leading to a gradual reduction in leaf stomatal conductance and photosynthetic carbon fixation^14^. This ultimately results in a decrease in mangrove forest biomass^67^.

Studies in Colombia have shown that carbon storage in mangrove forests’ AGB ranges from 37.9 to 102.1 Mg C ha^−1^^24,28^, with our study estimating it at 49.6 Mg C ha^−1^. These variations are influenced by the conservation level of mangrove forests. For instance, in Turbo, a town located in the Gulf of Urabá in the department of Antioquia, deforestation and sedimentation led to the lowest carbon estimate (37.9 Mg C ha^−1^), while the Rinconada sector of the Ciénaga Grande de Santa Marta in the department of Magdalena, the largest coastal wetland in Colombia, showed the highest value (102.1 Mg C ha^−1^), benefiting from freshwater input^28^. *Avicennia germinans* was the major contributor to AGB and carbon in both locations. Our study in the Mallorquin Swamp reported a carbon value of 49.6 Mg C ha^−1^, lower than estimates in Cispatá Bay in the Colombia’s Caribbean (64.85 Mg C ha^−1^) and Bahia Málaga in the Colombia’s Pacific coast (71.9 Mg C ha^−1^). These differences may be due to coastal geography and climate^88^. The Pacific coast’s deltas and high precipitation favor carbon storage, contrasting with the Caribbean’s drier climate and fewer mangroves in wetlands and lagoons^28,89^, as seen in the Mallorquin Swamp.

Notably, these carbon figures far exceed other estimates worldwide. For instance, Mexico’s Sian Ka’an Biosphere Reserve has the lowest estimation at 2.6 Mg C ha^−1^^90^,while Yap Island in Micronesia boasts the highest estimation globally at 434.8 Mg C ha^−1^^1^, showcasing the significant variability in mangrove carbon reserves across different regions.

The construction of the training wall on the Magdalena River significantly altered currents and sediment deposition^30^. The Mar station, with medium salinity is the geologically newest formation product of those shifts after the construction of the training wall^91^. Thus, an alternative explanation to the differences in vegetation structural composition may be the recent geological history of the area. We encourage further research in the dynamic nature of this system and how these anthropogenic changes may influence mangrove forests and carbon stocks.

Although our study provides a first approximation to the carbon stocks and ecological dynamics of this important urban mangrove, there is still needed to account for some potential biases in our study. First, we stress that the sample size in our study could be increased by adding additional transects in each station to capture the high mangrove variability. Also, incorporating larger and permanent plots will allow us to pinpoint additional mechanisms that drive the carbon balance and vegetation dynamics in this ecosystem. Furthermore, we also encourage further studies that focus on differences in soil nutrients microbial communities and potential contaminants as determinants of carbon stocks and tree diversity across the swamp. All of these have been previously recognized as important determinants of mangrove ecosystem dynamics^92,93^.

Our study in the Mallorquin Swamp has provided crucial insights into the complex interplay between environmental factors and mangrove ecosystems. While we focused specifically on pH and salinity, which are known to significantly influence mangrove physiology, species distribution, and soil processes, we recognize that anthropogenic drivers such as illegal settlements, deforestation, and infrastructure development can indirectly modify salinity regimes by disrupting hydrological connectivity and freshwater inflows^94^. These changes can, in turn, alter species composition and limit carbon accumulation in biomass. For example, land use change and human encroachment may intensify saline intrusion, altering hydrological patterns and reshaping the spatial distribution of more salt-tolerant mangrove species^95^. However, by isolating salinity as a key variable, we identified consistent patterns in mangrove composition and aboveground carbon, highlighting the ecosystem’s sensitivity to salinity, particularly in urban wetlands where human impacts intensify stressors. Future studies should explore a broader range of environmental drivers to better understand their impact on this mangrove community.

Despite these limitations, our findings provide significant contributions to the comprehensive scientific understanding of mangrove resilience and adaptive methods. The identification of species-specific responses to salinity, such as *Avicennia germinans* tolerance to low salinity and *Rhizophora mangle* adaptability to higher salinity, underscores the ecological complexity of mangrove ecosystems. This knowledge is essential for designing effective management and restoration plans that consider local environmental variations and species characteristics, supporting the long-term sustainability of mangrove habitats worldwide. For example, our study provides a first basis to understand which species should be used when restoring different areas of Mallorquin. This may change the traditional perspective in Mallorquin which has focused on restoring the ecosystem using mainly *Rhizophora mangle*. Instead, there should be a differential use of *Avicennia germinans*, *Rhizophora mangle* and *Laguncularia racemosa* according to the water and soil salinity.

Beyond its local relevance, our study offers valuable insights for informing broader conservation and climate policy frameworks. The identification of species-specific responses to soil salinity and their link to AGB carbon stocks can guide Colombia’s National Restoration Strategy 2023–2026 which aims to restore over 753,000 hectares by 2026, with a focus on strategic ecosystems such as mangroves and coastal wetlands^96^. Furthermore, this research aligns with Colombia’s climate commitments under the Paris Agreement, particularly its updated Nationally Determined Contribution (NDC), which pledges to reduce greenhouse gas emissions by 51% by 2030 and achieve carbon neutrality by 2050^97^. By identifying priority areas and appropriate species for restoration based on salinity conditions, our findings can also contribute to blue carbon initiatives and carbon offset strategies. Finally, this study supports the achievement of several United Nations Sustainable Development Goals (SDGs), particularly SDG 13 (Climate Action), SDG 14 (Life Below Water), and SDG 15 (Life on Land), by enhancing the scientific basis for restoring and conserving mangrove ecosystems in urban, climate-sensitive coastal areas^98^.

## Data Availability

The datasets used and analyzed during the current study are available from the corresponding author upon reasonable request.

## References

[1] Donato, D. C. et al. Mangroves among the most carbon-rich forests in the tropics. *Nat. Geosci.* **4**, 293–297 (2011).

[2] Kauffman, J. B., Donato, D. & Adame, M. F. *Protocolo Para La Medición, Monitoreo y Reporte de La Estructura, Biomasa y Reservas de Carbono de Los Manglares*. (2013). Retrieved from https://www.cifor-icraf.org/knowledge/publication/4386/ on 15 Mar 2020.

[3] Murdiyarso, D. et al. The potential of Indonesian mangrove forests for global climate change mitigation. *Nat. Clim. Chang.* **5**, 1089–1092 (2015).

[4] Kauffman, J. B. et al. Total ecosystem carbon stocks of mangroves across broad global environmental and physical gradients. *Ecol. Monogr.* **90**, 1–18 (2020).

[5] de Oliveira Gomes, L. E. et al. Ecosystem carbon losses following a climate-induced mangrove mortality in Brazil. *J. Environ. Manage.* **297**, 113381 (2021). 34325365 10.1016/j.jenvman.2021.113381

[6] Spalding, M. D. & Leal, M. *The State of the World’s Mangroves 2021*. (2021).

[7] Monga, E., Mangora, M. M. & Trettin, C. C. Impact of mangrove planting on forest biomass carbon and other structural attributes in the Rufiji Delta, Tanzania. *Glob. Ecol. Conserv.* **35**, e02100 (2022).

[8] Fourqurean, J. *et al. Carbono Azul: Métodos Para Evaluar Las Existencias y Los Factores de Emisión de Carbono En Manglares, Marismas y Pastos Marinos*. (2019). Retrieved from http://thebluecarboninitiative.org/ on 17 Jan 2022.

[9] Travieso-Bello, A. C., Moreno-Casasola, P. & Campos, A. Efecto de diferentes manejos pecuarios sobre el suelo y la vegetación en humedales transformados a pastizales. *Interciencia* **30**, 12–18 (2005).

[10] Flores-Verdugo, F. et al. La topografía y el hidroperíodo: Dos factores que condicionan la restauración de los humedales costeros. *Boletín la Soc. Botánica México* **80**, 33–47 (2007).

[11] Alsumaiti, T. & Shahid, S. Comprehensive analysis of mangrove soil in eastern lagoon national park of abu dhabi emirate. *Int. J. Bus. Appl. Soc. Sci.* **4**, 39–56 (2018).

[12] Parida, A. K. & Das, A. B. Salt tolerance and salinity effects on plants: A review. *Ecotoxicol. Environ. Saf.* **60**, 324–349 (2005). 15590011 10.1016/j.ecoenv.2004.06.010

[13] Sitoe, A. A., Mandlate, L. J. C. & Guedes, B. S. Biomass and carbon stocks of Sofala Bay mangrove forests. *Forests* **5**, 1967–1981 (2014).

[14] Rodríguez-Rodríguez, J. A., Mancera Pineda, J. E., Melgarejo, L. M. & Medina Calderón, J. H. Functional traits of leaves and forest structure of neotropical mangroves under different salinity and nitrogen regimes. *Flora* **239**, 52–61 (2018).

[15] Agraz-Hernández, C. M. et al. Carbon stocks in a mangrove ecosystem in northern Mexico: Environmental changes for 35 years. *Rev Mex. Biodivers.*10.22201/ib.20078706e.2020.91.3110 (2020).

[16] Ahmed, S. et al. Mangrove tree growth is size-dependent across a large-scale salinity gradient. *For. Ecol. Manage.* **537**, 120954 (2023).

[17] Boto, K. G., Bunt, J. S. & Wellington, J. T. Variations in mangrove forest productivity in northern Australia and Papua New Guinea. *Estuar. Coast. Shelf Sci.* **19**, 321–329 (1984).

[18] Joshi, H. G. & Ghose, M. Community structure, species diversity, and aboveground biomass of the Sundarbans mangrove swamps. *Trop. Ecol.* **55**, 283–303 (2014).

[19] Yoshikai, M. et al. Predicting mangrove forest dynamics across a soil salinity gradient using an individual-based vegetation model linked with plant hydraulics. *Biogeosciences* **19**, 1813–1832 (2022).

[20] Branoff, B. L. & Martinuzzi, S. The structure and composition of puerto rico’s urban mangroves. *Forests* **11**, 1–23 (2020). 10.3390/f11101119 PMC7751619 33365113

[21] Allais, L. et al. Salinity, mineralogy, porosity, and hydrodynamics as drivers of carbon burial in urban mangroves from a megacity. *Sci. Total Environ.* **912**, 168955 (2024). 38056642 10.1016/j.scitotenv.2023.168955

[22] Islam, M. A. et al. Dominant species losing functions to salinity in the Sundarbans Mangrove Forest, Bangladesh. *Reg. Stud. Mar. Sci.* **55**, 102589 (2022).

[23] Gallo-Vélez, D., Restrepo, J. C. & Newton, A. A socio-ecological assessment of land-based contamination and pollution: The Magdalena delta, Colombia. *Front. Mar. Sci.* **9**, 1–25 (2022).35450130

[24] Blanco-Libreros, J. F., Ortiz-Acevedo, L. F. & Urrego, L. E. Reservorios de biomasa aérea y de carbono en los manglares del golfo de Urabá (Caribe colombiano). *Actual. Biológicas* **37**, 131–141 (2015).

[25] Monsalve, A. & Ramírez, G. Caracterización de la estructura y contenido de carbono de los bosques de manglar en el área de jurisdicción del Consejo comunitario La Plata, Bahía Málaga, Valle del Cauca. **57**, 80 (2015).

[26] Yepes, A. et al. Ecuaciones alométricas de biomasa aérea para la estimación de los contenidos de carbono en manglares del Caribe Colombiano. *Rev. Biol. Trop.* **64**, 913–926 (2016). 29451977

[27] Bolivar, J. M., Gutierrez-Velez, V. H. & Sierra, C. A. Carbon stocks in aboveground biomass for Colombian mangroves with associated uncertainties. *Reg. Stud. Mar. Sci.* **18**, 145–155 (2018).

[28] Perdomo-Trujillo, L. Biomasa y producción radicular en manglares de cuenca neotropicales a lo largo de una trayectoria de restauración y su contribución a las reservas de carbono en el ecosistema. (Universidad Nacional de Colombia, 2020).

[29] Fuentes Delgado, J. E. La desaparición de las islas: Cambios ambientales en el delta del río Magdalena desde la cartografía histórica. *Hist. y Espac.* **18**, 0--3 (2022).

[30] Torres-Marchena, C. A., Flores, R. P. & Aiken, C. M. Impacts of training wall construction on littoral sedimentation under seasonal flow variability and sea-level rise: A case study of the Magdalena River (Colombia). *Coast. Eng.* **183**, 104306 (2023).

[31] INVEMAR. *Actualización y Ajuste Del Diagnóstico y Zonificación de Los Manglares de La Zona Costera Del Departamento Del Atlántico, Caribe Colombiano*. (2005). Retrieved from https://www.invemar.org.co/redcostera1/invemar/docs/Diag-Zonific.pdf/ on 10 Jan 2020.

[32] Garcés-Ordóñez, O., Ríos-Mármol, M. & Vivas-Aguas, J. L. *Evaluación de La Calidad Ambiental de Los Manglares de La Ciénaga Mallorquín, Departamento Del Atlántico*. (2016).

[33] Pineda, F. et al. Community preferences for participating in ecotourism: A case study in a coastal lagoon in Colombia. *Environ. Challenges* **11**, 100713 (2023).

[34] Berrocal, J. C., Ortega, A. M., Reales, R., González, S. & Calderón, R. Contaminación en la Ciénaga de Mallorquín: una perspectiva sociojurídica. *Educ. Socioambiental. Acción Present.* 231–262 (2018).

[35] Restrepo, J. D. & López, S. A. Morphodynamics of the Pacific and Caribbean deltas of Colombia, South America. *J. South Am. Earth Sci.* **25**, 1–21 (2008).

[36] Benavides, L. Análisis de la influencia de la calidad del agua del Arroyo Leon en la calidad del agua de la Cienaga de Mallorquín. (Universidad del Norte, 2019).

[37] Universidad del Norte. *Análisis Sobre El Manejo Integrado Del Recurso Hídrico En La Ciénaga de Mallorquín.* (2005). Retrieved from https://www.yumpu.com/xx/document/view/56802314/analisis-sobre-el-manejo-integrado-del-recurso-hidrico-en-la-cienaga-de-mallorquin on 13 Dec 2021.

[38] Navarrete, M. De las transformaciones ecológicas a los medios de vida de los pescadores artesanales en lagunas costeras: Estudio de caso del barrio de Las Flores en la Ciénaga de Mallorquín, Barranquilla, departamento del Atlántico, Colombia (1970 - 2020). (2022).

[39] Fuentes-Gandara, F., Pinedo-Hernández, J., Gutiérrez, E., Marrugo-Negrete, J. & Díez, S. Heavy metal pollution and toxicity assessment in Mallorquin swamp: A natural protected heritage in the Caribbean Sea, Colombia. *Mar. Pollut. Bull.* **167**, 112271 (2021). 33780754 10.1016/j.marpolbul.2021.112271

[40] Maldonado-Román, M., Jiménez-Collazo, J., Malavé-Llamas, K. & Musa-Wasill, J. C. Mangroves and their response to a heavy metal polluted wetland in the North Coast of puerto rico. *J. Trop. life Sci.* **6**, 210–218 (2016).

[41] Nguyen, A. et al. Long-term heavy metal retention by mangroves and e ff ect on its growth: A field inventory and scenario simulation. *Int. J. Environ. Res. Public Health* **17**, 1–24 (2020). 10.3390/ijerph17239131 PMC7729893 33297439

[42] Ministerio de Ambiente, V. y D. T. *Decreto 3888 de 2009*. (2009).

[43] Castro, E., Pinedo, J., Marrugo, J. & León, I. Retention and vertical distribution of heavy metals in mangrove sediments of the protected area swamp of Mallorquin, Colombian Caribbean. *Reg. Stud. Mar. Sci.* **49**, 102072 (2022).

[44] Villadiego, K. & Velay-Dabat, M. A. Outdoor thermal comfort in a hot and humid climate of Colombia: Afield study in Barranquilla. *Build. Environ.* **75**, 142–152 (2014).

[45] Merecí-Guamán, J., Casanoves, F., Delgado-Rodríguez, D., Ochoa, P. & Cifuentes-Jara, M. Impact of shrimp ponds on mangrove blue carbon stocks in Ecuador. *Forests* **12**, 1–14 (2021).

[46] Fraiz-Toma, A. et al. Carbon Stocks in Two Aquatic Marshes on the Caribbean and Pacific Coast of Panama. *Climate* **12**, 1–14 (2024).

[47] Chave, J. et al. Tree allometry and improved estimation of carbon stocks and balance in tropical forests. *Oecologia* **145**, 87–99 (2005). 15971085 10.1007/s00442-005-0100-x

[48] Chave, J. et al. Towards a worldwide wood economics spectrum. *Ecol. Lett.* **12**, 351–366 (2009). 19243406 10.1111/j.1461-0248.2009.01285.x

[49] Zanne, A. *et al.* Global Wood Density Database. *DRYAD Digital Repository* https://datadryad.org/stash/dataset/doi:10.5061/dryad.234 (2009).

[50] Anderson, M. J. & Walsh, D. C. I. PERMANOVA, ANOSIM, and the Mantel test in the face of heterogeneous dispersions: What null hypothesis are you testing?. *Ecol. Monogr.* **83**, 557–574 (2013).

[51] Hill, M. O. Diversity and Evenness: A Unifying Notation and Its Consequences. *Ecology* **54**, 427–432 (1973).

[52] Jost, L. Entropy and diversity. *Oikos* **113**, 363–375 (2006).

[53] Legendre, P. & Legendre, L. *Numerical Ecology*. *Elsevier 3rd Edition* vol. 24 (2012).

[54] Dennis, B., Ponciano, J. M., Taper, M. L. & Lele, S. R. Errors in Statistical Inference Under Model Misspecification: Evidence, Hypothesis Testing, and AIC. *Front. Ecol. Evol.* **7**, 372 (2019). 34295904 10.3389/fevo.2019.00372 PMC8293863

[55] Satheeshkumar, P. & Khan, B. A. Seasonal Variations in Physico-Chemical Parameters of Water and Sediment Characteristics of Pondicherry Mangroves. *African J. Basic Appl. Sci.* **1**, 36–43 (2009).

[56] Vijaya Kumar, K. & Kumara, V. Seasonal variations in physico-chemical parameters of mangrove water, Kundapur, southwest coast of India. *J. Aquat. Biol. Fish.* **2**, 852–858 (2014).

[57] Flores-De-Santiago, F., Kovacs, J. M. & Flores-Verdugo, F. Seasonal changes in leaf chlorophyll a content and morphology in a sub-tropical mangrove forest of the Mexican Pacific. *Mar. Ecol. Prog. Ser.* **444**, 57–68 (2012).

[58] Smith, T. J. Forest structure. *Trop. Mangrove Ecosyst.* **41**, 101–136 (1992).

[59] Woodroffe, C. Mangrove Sediments and Geomorphology. in *Tropical Mangrove Ecosystems* vol. 41 (Coastal and Estuarine Studies, 1992).

[60] Cardona, P. & Botero, L. Soil characteristics and vegetation structure in a heavily deteriorated mangrove forest in the Caribbean Coast of Colombia. *Biotropica* **30**, 24–34 (1998).

[61] Rodríguez-Ramírez, A., Nivia-Ruíz, J. & Garzón-Ferreira, J. Características estructurales y funcionales del manglar de Avicennia germinans en la Bahía de Chengue (Caribe colombiano). *Boletín Investig. Mar. y Costeras* **33**, 223–244 (2004).

[62] Yanez-Espinosa, L. & Flores, J. A Review of Sea-Level Rise Effect on Mangrove Forest Species: Anatomical and Morphological Modifications. *Glob. Warming Impacts - Case Stud. Econ., Hum. Health, Urban Nat. Environ.*10.5772/24662 (2011).

[63] Lonard, R. I., Judd, F. W., Summy, K. R., Deyoe, H. & Stalter, R. The biological flora of coastal dunes and wetlands: Avicennia germinans (L.) L. *J. Coast. Res.* **0**, (2017).

[64] Yáñez-Espinosa, L., Angeles, G., López-Portillo, J. & Barrales, S. Variación anatómica de la madera de Avicennia germinans en la Laguna de La Mancha, Veracruz, MÉxico. *Bot. Sci.* **85**, 7–15 (2009).

[65] Gonzalez Rodriguez, H. et al. Comparative Morphology and Anatomy of Few Mangrove Species in Sundarbans, West Bengal, India and its Adaptation to Saline Habitat. *Int. J. Bio-Resource Stress Manag.* **3**, 1–17 (2012).

[66] López, B., Barreto, M. & Conde, J. Caracterización de los manglares de zonas semiáridas en el noroccidente de Venezuela. *Interciencia* **36**, 888–893 (2011).

[67] Suárez, N. & Medina, E. Salinity effect on plant growth and leaf demography of the mangrove, Avicennia germinans L. *Trees - Struct. Funct.* **19**, 721–727 (2005).

[68] Torres-Duque, J. Complejidad Estructural Aérea de Bosques de Manglar y su Relación con Contenido de Carbono Azul en Suelos. (2019). Retrieved from https://repositorio.unal.edu.co/handle/unal/75951 on 20 Dec 2021.

[69] INVEMAR. *Monitoreo de Las Condiciones Ambientales y Los Cambios Estructurales y Funcionales de Las Comunidades Vegetales y de Los Recursos Pesqueros Durante La Rehabilitación de La Ciénaga Grande de Santa Marta*. (2018).

[70] Soares, M. L. G., Chaves, F. D. O., Corrêa, F. M. & da Silva Júnior, C. M. G. Diversidade estrutural de bosques de mangue e sua relação com distúrbios de origem antrópica: o caso da Baía de Guanabara (Rio de Janeiro). *Anuário do Inst. Geociências* **26**, 101–116 (2003).

[71] De Sousa Paula, A. L., De Sousa Lima, B. K. & Camargo Maia, R. Recuperação de um manguezal degradado no ceará através da produção de mudas de Laguncularia racemosa (L.) C.F. Gaertn. (Combretaceae) E Avicennia sp. Stapf ex Ridl (Acanthaceae). *Rev. Arvore* **40**, 377–385 (2016).

[72] Bai, J. et al. The linkages between stomatal physiological traits and rapid expansion of exotic mangrove species (Laguncularia racemosa) in new territories. *Front. Mar. Sci.* **10**, 1136443 (2023).

[73] Chandrasekara, C. M. K. N. K., Weerasinghe, K. D. N., Pathirana, S. & Piyadasa, R. U. K. Mangrove Diversity Across Salinity Gradient in Negombo Estuary-Sri Lanka. *Environ. Geogr. South Asia*10.1007/978-4-431-55741-8 (2016).

[74] Krauss, K. W. et al. Environmental drivers in mangrove establishment and early development: A review. *Aquat. Bot.* **89**, 105–127. 10.1016/j.aquabot.2007.12.014 (2008).

[75] Lopes, D. M. S., Tognella, M. M. P., Falqueto, A. R. & Soares, M. L. G. Salinity variation effects on photosynthetic responses of the mangrove species rhizophora mangle l. Growing in natural habitats. *Photosynthetica* **57**, 1142–1155 (2019).

[76] Ellison, A. M. & Farnsworth, E. J. Seedling survivorship, growth, and response to disturbance in Belizean mangal. *Am. J. Bot.* **80**, 1137–1145 (1993).

[77] Tovilla, C. & Orihuela, D. E. Floración, establecimiento de propágulos y supervivencia de Rhizophora mangle L. en el manglar de Barra de Tecoanapa, Guerrero, México. *Madera y Bosques* **8**, 89–102 (2002).

[78] Monroy-Torres, M., Flores-Verdugo, F. & Flores-de-Santiago, F. Crecimiento de tres especies de mangle subtropical en respuesta a la variabilidad en el hidroperiodo en un tanque experimental. *Ciencias Mar.* **40**, 263–275 (2014).

[79] Evans, L. S., Okawa, Y. & Searcy, D. G. Anatomy and morphology of red mangrove (Rhizophora mangle) plants in relation to internal airflow. *J. Torrey Bot. Soc.* **132**, 537–550 (2005).

[80] Feng, X. et al. Molecular adaptation to salinity fluctuation in tropical intertidal environments of a mangrove tree Sonneratia alba. *BMC Plant Biol.* **20**, 1–14 (2020). 32321423 10.1186/s12870-020-02395-3 PMC7178616

[81] Dittmann, S. et al. Effects of Extreme Salinity Stress on a Temperate Mangrove Ecosystem. *Front. For. Glob. Chang.* **5**, 1–18 (2022).

[82] Mizanur Rahman, M. et al. Carbon stock in the Sundarbans mangrove forest: spatial variations in vegetation types and salinity zones. *Wetl. Ecol. Manag.* **23**, 269–283 (2015).

[83] Bache, B. The role of calcium in buffering soils. *Plant, Cell Environ.* **7**, 391–395 (1984).

[84] Somridhivej, B. & Boyd, C. E. Likely effects of the increasing alkalinity of inland waters on aquaculture. *J. World Aquac. Soc.*10.1111/jwas.12405 (2017).

[85] Molina, A., Molina, C., Giraldo, L. & Barrera, R. Caracteristicas estratigraficas y morfodinamicas de la franja litoral Caribe Colombiana (sector Barranquilla (Bocas de Ceniza) - flecha de Galerazamba). *Bol. Investig. Mar. y Costeras* **28**, 61–94 (1999).

[86] Medivelso, D., Carvajal, H. & Pinzon, L. Estudio geomorfológico del sector comprendido entre Bocatocino (Atlántico) y Ciénaga (Magdalena). *Boletín Científico CIOH* **2**, 140 (2010).

[87] Perri, S., Entekhabi, D. & Molini, A. Plant osmoregulation as an emergent water-saving adaptation. *Water Resour. Res.* **54**, 2781–2798 (2018).

[88] Blanco-Libreros, J. F. & Álvarez-León, R. Mangroves of Colombia revisited in an era of open data, global changes, and socio-political transition: Homage to Heliodoro Sánchez-Páez. *Rev. la Acad. Colomb. Ciencias Exactas, Fis. y Nat.* **43**, 84–97 (2019).

[89] Pérez, M., Navarro, M. & Saborío, M. *Adaptación Basada En Ecosistemas: Los Manglares*. (2018).

[90] Adame, M. F. et al. Carbon Stocks of Tropical Coastal Wetlands within the Karstic Landscape of the Mexican Caribbean. *PLoS ONE* **8**, e56569 (2013). 23457583 10.1371/journal.pone.0056569 PMC3572964

[91] Restrepo, J. C. et al. Siltation on a highly regulated estuarine system: The Magdalena River mouth case (Northwestern South America). *Estuar. Coast. Shelf Sci.* **245**, 107020 (2020).

[92] Holguin, G., Vazquez, P. & Bashan, Y. The role of sediment microorganisms in the productivity, conservation, and rehabilitation of mangrove ecosystems: An overview. *Biol. Fertil. Soils* **33**, 265–278 (2001).

[93] Bayen, S. Occurrence, bioavailability and toxic effects of trace metals and organic contaminants in mangrove ecosystems: A review. *Environ. Int.* **48**, 84–101 (2012). 22885665 10.1016/j.envint.2012.07.008

[94] Alongi, D. M. Impact of global change on nutrient dynamics in Mangrove Forests. *Forests* **9**, 1–13 (2018).

[95] Mukhopadhyay, A., Wheeler, D., Dasgupta, S., Dey, A. & Sobhan, I. Mangrove Spatial Distribution in the Indian Sundarbans: Predicting Salinity-Induced Migration in a Changing Climate. *J. Manag. Sustain.* **9**, 1 (2019).

[96] Ministerio de Ambiente y Desarrollo Sostenible. *Estrategia Nacional de Restauración 2023 - 2026*. (2024). Retrieved from https://www.patrimonionatural.org.co/wp-content/uploads/2024/10/ENR-2023-2026_05MAY2024.pdf on 13 Apr 2025.

[97] Gobierno de Colombia. *Estrategia Climática de Largo Plazo de Colombia E2050 Para Cumplir Con El Acuerdo de París*. (2021). Retrieved from https://www.minambiente.gov.co on 14 Apr 2025.

[98] United Nations. *Transforming Our World: The 2030 Agenda for Sustainable Development*. (2015). Retrieved from https://sdgs.un.org/sites/default/files/publications/21252030%20Agenda%20for%20Sustainable%20Development%20web.pdf on 14 Apr 2025.

